# Does the dual circulation coupling synergy facilitate firm survival?——Evidence from China

**DOI:** 10.1371/journal.pone.0290448

**Published:** 2023-08-21

**Authors:** Lijun Ran, Xin Li, Kang Wang, Huihui Liu

**Affiliations:** School of Mathematics and Statistics, Beijing Technology and Business University, Beijing, China; Guangxi Normal University, CHINA

## Abstract

The “dual circulation” development pattern is a major economic strategy proposed by China. Using samples gathered from firms located in the Haidian Science and Technology Park in Zhongguancun between 2013 and 2019, this paper constructs an index system for measuring the degree of synergy of the dual circulation coupling of the firms, and then uses the Cloglog survival model to empirically verify its effect on the survival of firms. It is found that the dual circulation coupling synergy of firms significantly contributes to firm survival, a finding that still holds after robustness tests. Mechanism tests show that firm dual circulation coupling synergies contribute to firm survival by promoting firm innovation, market demand, and resource allocation efficiency. Further analysis reveals that the coupled synergy of the dual circulation of firms contributes more significantly to the survival of growing and mature firms, MSMEs, private firms and non-start-up firms. This paper is the first to measure dual circulation. It provides micro-theoretical mechanisms and relevant empirical evidence for the dual circulation coupling synergy for firm survival.

## Introduction

The survival of firms is fundamental to their development. The American magazine, Fortune, reports that the average U.S. small to medium enterprise (SME) lasts fewer than seven years and the average large firm survives less than 40 years. In China, SMEs survive 2.5 years and conglomerates 7–8 years. Firm survival is determined not only by the entrepreneur’s success or failure, but also by the promotion of the new development pattern of the dual circulation and social stability. Due to trade frictions, Chinese firms have experienced diminishing overseas demand, the recycling and transfer of industrial chains by industrialized countries, and insufficient local effective demand. After the new crown pneumonia pandemic has made it harder for firms to resume work, and the survival rate of small and micro firms has dropped by 11.81% (Liao et al., 2021) [1], the problem of firm survival is further exacerbated. Dual circulation, the utilization of two markets and resources by firms, provides opportunities for the survival of firms, reflecting the integration of firms into the two markets, creating an equilibrium where “the east is not bright and the west is bright”. In the face of developed countries carrying out technological embargo, the innovation capacity of firms will be inhibited in the short term. In the long run, will push firms to improve their innovation capacity. Sino-US trade friction forces Chinese firms to pursue internal circulation and to develop the domestic market in terms of operation, production and demand, and the huge domestic market demand potential expands the space for firm survival. Firm dual circulation coupling synergy prompts the free flow of production factors and enhances the efficiency resource allocation, which will enhance the probability of firm survival.

Current research focuses on the effects of the following factors on firms: firm age (Honjo, 2000; Esteve-Pérez et al., 2008) [2, 3]; financing constraints (Audia et al., 2006) [4]; innovation (Kimura et al., 2003; Bayus et al., 2007) [5, 6]; exports (Dai et al., 2016) [7]; government subsidies (Mao et al., 2015) [8]; FDI (Chen et al., 2021) [9]; firm type (Görg et al., 2003; Colombo et al., 2000) [10, 11].

Two other papers are closely related to this paper: Zhao et al. (2022) and Lin et al. (2022). Zhao et al. (2022) [12] constructed a measure of economic inner cycle with consumption and production, a measure of economic outer cycle with investment, import-export trade, and technology introduction, and on this basis measured the degree of coupling synergy between the “inner cycle” and “outer cycle” of China’s economy. It is found that the coupling synergy degree between the "inner cycle" and the "outer cycle" of China’s economy show a steady upward trend. Using domestic demand and exports as proxy variables for the internal and external circulation, Lin et al. (2022) [13] investigate how the “home appliances to the countryside” policy caused by internal circulation, promotes external circulation. The increase in domestic demand for home appliances is found to significantly promote exports.

However, existing literature does not examine the impact of corporate dual circulation coupling synergy on firm survival. Compared with existing studies, this paper makes three marginal contributions: (1) provision of an elaboration of the economic cycle at micro levels and definition of firm dual circulation; (2) construction of an index system for measuring the dual circulation of firms and measurement of the coupling synergy degree; (3) exploration of the relationship between coupled synergy and the survival of firms by using the Cloglog survival analysis model. It is found that coupled synergy can improve the survival rate of firms, and the key mechanisms identified are market demand effect, innovation effect and resource allocation effect. The results provide a reference for improving the survival rate of firms in the new development pattern of dual circulation.

## Theoretical analysis and research hypothesis

### Direct effect of the firm dual circulation coupling synergy on firm survival

The capital cycle reveals that firm capital goes through three stages: purchase—production—sale. Before a firm can return to the starting point, it must reasonably allocate the three forms of capital in time and space. Otherwise, the entire capital cycle will stagnate. There is a micro and macro relationship between individual capital and social capital. The smoothness of the circulation will affect the overall cycle. Therefore, the key to the “dual circulation” development paradigm, in which domestic and overseas markets reinforce each other, with the domestic market as the mainstay, is the firm circulation. The capital cycle in the context of globalization is not limited to one country; the acquisition of production factors, production activities and consumption, include both domestic and international markets. The internal cycle uses domestic factors to provide products for the domestic market, and the external cycle uses international factors to provide products for the foreign market (Li, 2021) [14]. As the primary body of the market economy, firms must engage in both cycles. In this paper, the internal cycle of an firm is defined as the capital cycle in which the firm’s "purchases—production—sales" all take place in the domestic market, and the capital cycle of "money capital—production capital—commodity capital" is completed sequentially. The firm’s international cycle is defined as its capital circulation through the export of money, production, and commodity capital in international markets. the domestic and international cycles of firm are called “firm dual circulation”.

Previous studies show that since China’s reform and opening up, internal circulation and external circulation have been inextricably linked (Liu, 2020) [15]. Few existing studies examine firm-level dual circulation coupling synergies. This study reviews a significant body of literature on firm usage of internal and external marketplaces for domestic sales, imports, and exports. According to Melitz (2003) [16], exporting firms face two distinct markets: domestic and export, and high productivity firms prefer to export while low productivity firms choose to sell domestically. Some scholars argue that market segmentation leads to export trade costs being lower than domestic trade costs. Therefore, low productivity firms choose to export (Zhang et al., 2010) [17], implying that domestic sales and exports are independent of one another.

However, the rigidity of the factor market leads to firm capacity constraints in the short term. In the face of positive external demand, firms must reduce domestic sales in order to increase exports, and exports and domestic sales have a substitution relationship (Blum et al., 2013) [18]. According to Berman et al. (2015) [19], domestic sales and exports are complementary; exporting permits firms to communicate with international markets and use “learning by doing” to keep improving their technology, which assists them in the stage of increasing returns to scale, and there is a significant “local market effect”. Krugman (1987) [20] argues that import liberalization squeezes the market space of local firms and so there is a substitution relationship between domestic sales and imports, thus imports can stimulate the productivity growth of local firms. Contrastingly, Shimomura et al. (2012) [21] argue that imports and domestic sales can promote productivity by adjusting the allocation of resources in the market and that there is an overall positive relationship between the two. Overseas R&D has helped with China’s technical advancement; import competition can spur innovation, and two-way trade can help firms to survive (Chen et al., 2012) [22]. Based on the abovementioned theories, this paper contends that there is a mutually beneficial and interdependent relationship between a firm’s domestic sales and imports and exports, implying a synergistic relationship between firms’ dual circulation, consistent with the relationship between the macro domestic and international dual circulation.

Whilst existing literature has studied the factors influencing firm survival, there exists little research on the synergy of two markets and two resources, both internal and external. One aspect is that market segmentation hinders a firm’s domestic market expansion and motivates them to export, which thus objectively increases the scale of foreign sales and makes Chinese firm exports oppose the “domestic demand-driven export model” (Yi et al., 2018) [23]. The segmentation of domestic and international markets makes trade integration insufficient in China. This hinders the continuous expansion of market scale and market space by making it difficult for firms to use two markets and resources to effectively promote development, and instead increases the probability of market exit.

The current foreign market is subject to negative demand fluctuations. Export to domestic sales provides a possibility to replace the international market. However, firms exporting products to domestic sales is not an abandoned international market, but to support marketable export products to develop the domestic market, to help export firms to survive.Market size has a large impact on industrial spatial agglomeration, and opening up to the outside world can stimulate firms to expand into foreign markets and promote the integration of domestic and foreign markets. Chinese firms can learn about foreign advanced technology through the international market and obtain the “export learning effect”, which improves the technical level of products and promotes a sustainable business (Qiu et al., 2012) [24]. Firms can fully exploit the comparative advantages of raw material supply in internal and external markets in order to reduce procurement and production costs, reducing the risk of survival of the firm. Therefore, we formulate Hypothesis 1:

H1: Firm dual circulation coupling synergy has a catalytic effect on firm survival.

### Mechanisms by which firm dual circulation coupling synergy affects firm survival

With the continuous integration of internal and external markets, firms face fierce competition in production and operation. They must therefore gain survival space through continuous innovation, market expansion, and optimal resource allocation.

#### Innovation effects

According to Esteve (2008) [3], firms with abundant resources are more competitive and risk-resistant. Innovation capability is at the core of a firm’s internal resources; the ability to survive in the market is influenced by a firm’s own innovation behavior, and those firms that survive are significantly more innovative than exiting firms. Firm dual circulation coupling synergy can stimulate firm innovation by expanding market demand and reducing innovation costs, which in turn promotes firm survival. Firm dual circulation coupling synergy can help fully exploit the similar demand of “two markets”, expand the scale of sales. Stimulate firm innovation through “demand induced”, to create products with higher quality and technology content (Zweimüller et al., 2005; Desmet et al., 2010) [25, 26], and realize the “dual market incentive of internal and external circulation” for firm innovation (Qing et al., 2021) [27]. Firm dual circulation coupling synergy can simultaneously respond to the multi-level demands of both domestic and international markets and stimulate the speed of innovation, which leads to the production of diversified products, cultivation of new advantages in international competition, and creation of the “demand structure-induced innovation” effect (Qian et al., 2021) [28]. The greater the potential of domestic and international market demand, the lower the R&D cost of innovation amortized to each unit of product, and thus the greater the profit margin earned by engaging in innovation activities, which provides more financial support for innovation and helps to reduce the risk of technological innovation and improve a firm’s efficiency (Fan, 2007) [29] and survival rate. This leads us to Hypothesis 2:

H2: Firm innovation is an important mechanism for firm dual circulation coupling synergy used to ensure firm survival.

#### Market demand effect

Firm dual circulation coupling synergy refers to the expansion of the market size. The domestic markets allow firms to gain agglomeration economic advantages, which facilitates communication and learning of market information and technology among firms. In turn, this helps firms maintain their market share and required market space. Firm dual circulation coupling synergy not only meets the demand of foreign consumers, but also shares the plight of insufficient domestic demand, accelerates the circulation of corporate capital, stabilizes corporate capital flow, eases corporate financing constraints, and expands the market scale and therefore, the survival space of firms. Simultaneously, access to the international markets provides firms with the opportunity to obtain advanced foreign technology and management experience, which effectively spreads their innovation and investment risk. Firm dual circulation coupling synergy also allows firms to distribute their products in two markets, allowing products with different life cycles to expand their sales to different markets and hedge firm’s risk (Agarwal et al., 2002) [30]. With regards to developing potential markets, firm dual circulation coupling synergy, which is where a firm markets its domestic sales products to the international market and correspondingly introduces international market sales products to the domestic market, enables a firm to share the fixed cost of building a brand through brand extension. The formation of two markets that promote and support each other (Han et al., 2014) [31], play a complementary effect in the process of firm survival. Therefore, we formulate Hypothesis 3 as follows:

H3: Market demand is an important mechanism for firm dual circulation coupling synergy to promote firm survival.

#### Resource allocation effect

Firm dual circulation coupling synergy enables the free flow of production factors to realize the effective allocation of resources, reduce the risk of firm survival. Lower firm resource allocation efficiency causes efficiency and output loss, which is reflected in the difference in the marginal substitution rate of capital, labor and other factors among firms, and the deviation of resource allocation efficiency from the Pareto optimal state. The optimization of resource allocation can reduce the marginal cost of firms (Yi et al., 2018) [32], which in turn reduces their average cost and leads to improvements in firm profitability and expansion of firm survival space. The dual circulation coupling synergy provides a firm with global access advantages. For example, exporters have access to market orders, and the resources, knowledge, and information required for the production process via cross-border formal and informal links, as well as access to cheaper, more diverse, or higher-quality intermediate products. There is also the benefit of learning about new technologies and products. Achieving better resource allocation efficiency can modify firm production through marginal cost, avoiding firm under-capacity or over-capacity (Bian et al., 2021) [33], boosting firm market competitiveness, expanding firm anti-risk ability, and increasing firm survival chance. Therefore, we formulate Hypothesis 4 as follows:

H4: Resource allocation efficiency is an important mechanism for firm dual circulation coupling synergy to promote firm survival.

**Fig 1:**
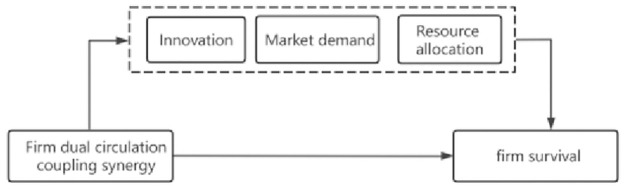
Theoretical framework. It shows the direct impact of firm dual circulation coupling synergy on firm survival,and the impact on the firm’s survival through the three mechanisms of innovation, market demand and resource allocation.

The theoretical framework is shown in Fig 1.

## Measuring firm dual circulation coupling synergy

### Construction of the indicator system

Previous scholars have studied dual circulation from different perspectives, but they mainly follow three measurement methods and indicators: (1) decomposition of value added using global input-output tables to measure the dependence of China’s domestic economic cycle (Huang et al., 2021; Chen et al., 2021; Chen, 2022; Ding et al., 2022) [34–37]; (2) establishment of an indicator system to measure the degree of coordination of internal and external circulation coupling at the macro level from the perspective of economic circulation (Zhao et al., 2022) [12]; (3) use of domestic demand and exports as proxy variables for the internal and external circulation of firms (Lin et al., 2022; Du et al., 2023) [13, 38]. However, there is no unified indicator system; in particular, there is a lack of measurement of the dual circulation of firms. Contrastingly, this paper constructs a system of indicators for measuring firm dual circulation from two dimensions: capital and human capital. The most essential feature of building a new development pattern of dual circulation is to achieve a high level of self-reliance and self-improvement, but firms are usually unable to create the necessary knowledge for innovation internally and therefore need to find knowledge from external resources (Ceccagnoli et al., 2014; Martini et al., 2017) [39, 40]. Innovation networks mainly acquire complementary knowledge and assets through formal and informal relationships, thus facilitating the creation and flow of knowledge in the network and providing firms with multiple knowledge and systemic resources (Wang, 2015) [41]. The “internal cycle” and “external cycle” of science and technology innovation (Yang, 2021) [42] are the principles and strategies based on the layout of domestic and foreign innovation for firms in the stage of catching up. Therefore, this paper incorporates the internal and external cycles of firm innovation into the measure of a firm’s dual circulation. Finally, using the two systems of internal and external circulation, this paper constructs an index system covering four secondary indicators, notably, innovation, human capital, industry-academia-research and financial flow, and several tertiary indicators, as shown in Table 1.

**Table 1:** Indicator system of the firm dual circulation system.

Total index	Dimension Layer	Sub-Level	Index Layer	Units
**Firm dual circulation system**	**Domestic Circulation**	**Independent Innovation (Ind-inno)**	Daily R&D expenditure within the firm	thousand yuan
			Internal expenses for all science and technology project funds	thousand yuan
			Number of domestic R&D facilities established	piece
			Expenditure on domestic R&D institutions	thousand yuan
			Number of domestic invention patent applications	piece
			Expenditure on the purchase of domestic technology	thousand yuan
			Number of domestic invention patents granted	piece
			Use of R&D funds from government	thousand yuan
			Number of valid invention patents granted in the territory	piece
			Domestic Trademark Registration	piece
			Revenue from domestic sales of new products	thousand yuan
		**Human Capital (Hum-cap)**	Number of practitioners with PhD and above in China	person
			Number of employees with a master’s degree in China	person
			Number of fresh graduates from universities absorbed	person
		**Industry-Academia-Research (Ind-acad-re)**	Commissioned outside units to carry out research and development of the funds for domestic research institutions and higher education expenditure	thousand yuan
			Commissioned outside units to carry out research and development of the funds for domestic higher education expenditure	thousand yuan
			Number of R&D institutions established outside Beijing	piece
		**Funds Liquidity (Funds-liq)**	Total tax deductions	thousand yuan
			Tax deductions for research and development expenses	thousand yuan
			High-tech firm tax deduction	thousand yuan
			Amount of innovation fund received this year	thousand yuan
			Amount of government procurement obtained	thousand yuan
			Subsidy income	thousand yuan
	**International Circulation**	**Technology Innovation (Tech-inno)**	Expenditure on the introduction of foreign technology	thousand yuan
			Expenditure on digestion and absorption of imported foreign technology	thousand yuan
			Number of patent applications in Europe, the United States and Japan	piece
			Number of PCT patent applications	piece
			Number of patents granted in Europe, America and Japan	piece
			Number of foreign licenses in the number of active patents	piece
			Number of valid invention patents granted abroad	piece
			Number of patents owned in Europe, America and Japan	piece
			Export revenue in new product sales revenue	thousand yuan
		**Human Capital (Hum-cap)**	Number of employees in Hong Kong, Macau, Taiwan and foreigners at the end of the period	person
			Number of foreign experts among the number of employees at the end of the period	person
			Number of returnees from abroad among the number of employees at the end of the period	person
		**Industry-Academia-Research (Ind-acad-re)**	Expenditures on research and development entrusted to an outside party	thousand yuan
			Number of overseas R&D institutions	piece
		**Funds Liquidity (Funds-liq)**	Amount of foreign direct investment	thousand yuan
			Total import and export	thousand yuan

### Coupling synergy model construction and data

#### Coupling synergy model

The coupling synergy degree measures the degree of coordinated development of subsystems within the system. We synthesize existing literature about the use of the coordination degree model, and construct a coordination degree model subsystem and integrated development level (Xu et al., 2021;Ge et al., 2020) [43, 47]. The coupled synergy degree model is composed of two or more subsystems, and the integrated development level of subsystems forms the core of the coupled coordination degree measurement.

Let the element *y*_*ij*_ in *y*_*ij*_ = (*i* = 1,2 …, *n*; *j* = 1,2 …., *m*) be the *j* indicator in subsystem *U*_*i*_, i.e., the ordinal parameter. *M*_*ij*_ and *m*_*ij*_ are the maximum and minimum values in subsystem *U*. The ordinal parameter can be divided into benefit ordinal parameter and cost ordinal parameter. When the benefit ordinal parameter as the original indicator is positive, the value of *y*_*ij*_ is larger and the degree of system order is higher. When the original indicator is negative, it’s larger and the degree of system order is lower. When the cost ordinal parameter is the original indicator is negative, the larger the value of *y*_*ij*_ is, the lower the degree of system order, and vice versa, the higher the degree of system order. To eliminate the effect of the magnitude, *y*_*ij*_ is normalized:

$$y_{ij}^{\prime }=\frac{y_{ij}-m_{ij}}{M_{ij}-m_{ij}},\;y_{ij}\;\text{is}\;\text{a}\;\text{positive}\;\text{indicator}$$

$$y_{ij}^{\prime }=\frac{M_{ij}-y_{ij}}{M_{ij}-m_{ij}},\;y_{ij}\;\text{is}\;\text{the}\;\text{inverse}\;\text{indicator}$$ (1)

The weight of the *j* ordinal covariate in the construction system *i* is:

$$x_{ij}=\frac{y_{ij}^{\prime }}{\sum_{i=1}^{n}\;y_{ij}^{\prime }}$$ (2)

Drawing on existing literature (Tang et al., 2018; Zhang et al., 2019) [44, 45], this paper uses information entropy to determine the weights. The information entropy of the *j* ordinal covariate in system *U*_*i*_ is:

$$e_{j}=-K\sum_{i=1}^{n}\;x_{ij}\;lnx_{ij},K=\frac{1}{lnn}$$ (3)

The information utility value of each indicator *d*_*j*_ depends on the difference between the information entropy of indicator *e*_*j*_ and 1. The greater the information utility value, the more important the evaluation, and the greater the weight:

$$d_{j}=1-e_{j}$$ (4)

The weights of the order parameter *j* is:

$$w_{j}=\frac{d_{j}}{\sum_{j=1}^{n}\;d_{j}}$$ (5)

The sequential covariates are weighted to obtain the integrated development level of the subsystem as:

$$u_{i}=\sum_{j=1}^{n}\;y_{ij}^{\prime }*w_{j}$$ (6)

Coupling synergy model refers to the synergistic development relationship between two or more subsystems that influence each other. This paper uses the capacity coupling coefficient model in physics to measure the coupling synergy between a firm’s inner and outer circulation systems (Valerie, 1996) [46]. The coupling degree model of the inner and outer circulation of a firm is constructed as:

$$C_{ab}^{t}=2\sqrt{u_{a}^{t}\cdot u_{b}^{t}}/(u_{a}^{t}+u_{b}^{t})$$ (7)

$C_{ab}^{t}$ is the coupling degree of the inner and outer cycles of the firm in year $t.\;u_{a}^{t}$ is the integrated development level of the inner cycle in year t, and $u_{a}^{t}$ is the integrated development level of the outer cycle in year *t*. If both $u_{a}^{t}$ and $u_{b}^{t}$ are low, then calculating only the coupling degree $C_{ab}^{t}$ will result in the pseudo-evaluation result whereby the two systems have a high degree of synergistic development when both development levels are lower. Therefore, we construct the coupling synergy model as follows:

$$D_{ab}^{t}=\sqrt{C_{ab}^{t}\cdot T_{ab}^{t}},\;T_{ab}^{t}=\alpha u_{a}^{t}+\beta u_{b}^{t}$$ (8)

$D_{ab}^{t}$ is the value of the coupling coordination degree of the internal and external circulation of the firm in year *t*, which takes the value range of [0, 1]. $T_{ab}^{t}$ is the comprehensive development level of the internal and external circulation of the firm in year *t*.*α* and *β* are coefficients to be determined (*α* + *β* = 1). This paper assumes that the internal and external circulation of the firm is equally important. Therefore, *α* and *β* are both set to 0.5 (Xu et al., 2021) [43].

Based on the coupling synergy degree value D of the firm’s dual circulation, the coupling synergy level of the two systems is split into four types (Ge et al., 2020) [47], as shown in Table 2.

**Table 2:** Judgment criteria of the coupling synergy relationship.

Coordination D value	0≤D<0.3	0.3≤D<0.5	0.5≤D<0.8	0.8≤D<1
**Basic Type**	Low	Moderate	High	Extreme

#### Data

This paper adopts the firms in Zhongguancun Haidian Park Science and Technology Park (ZGC) between 2013 and 2019 as the research object. This data set has the following three advantages. Firstly, ZGC firm is typical and representative and has a well-established network system. ZGC is the core region of China’s National Science and Technology Innovation Center, and has become a “barometer” reflecting China’s position in the global value chain (GVC). As a source of original innovation and a testing ground for institutional reform, the results of the “dual circulation” practice are relevant for policy evaluation and improvement directions. Secondly, ZGC relies on the capital city economy and follows the principle of two-way openness, which reflects coupling synergy and serves the new development pattern of national dual circulation. Thirdly, the data from firms located in ZGC have a long time span, and provide rich indicators. Data collected by the government with authority, it suitable for studying the impact of dual circulation coupling synergy on the survival of firms.

As there may be outliers in the sample, this paper follows Qianli Xie et al. (2008) [48] and eliminates the following: (1) samples with missing major financial indicators; (2) samples that do not comply with general accounting standards; (3)the continuous type indicators were all reduced in accordance with the bilateral exclusion of 1% each to eliminate the effect of outliers. Eventually arrive at 65125 samples.

#### Facts about the characteristics of firm dual circulation coupling synergy

**Fig 2:**
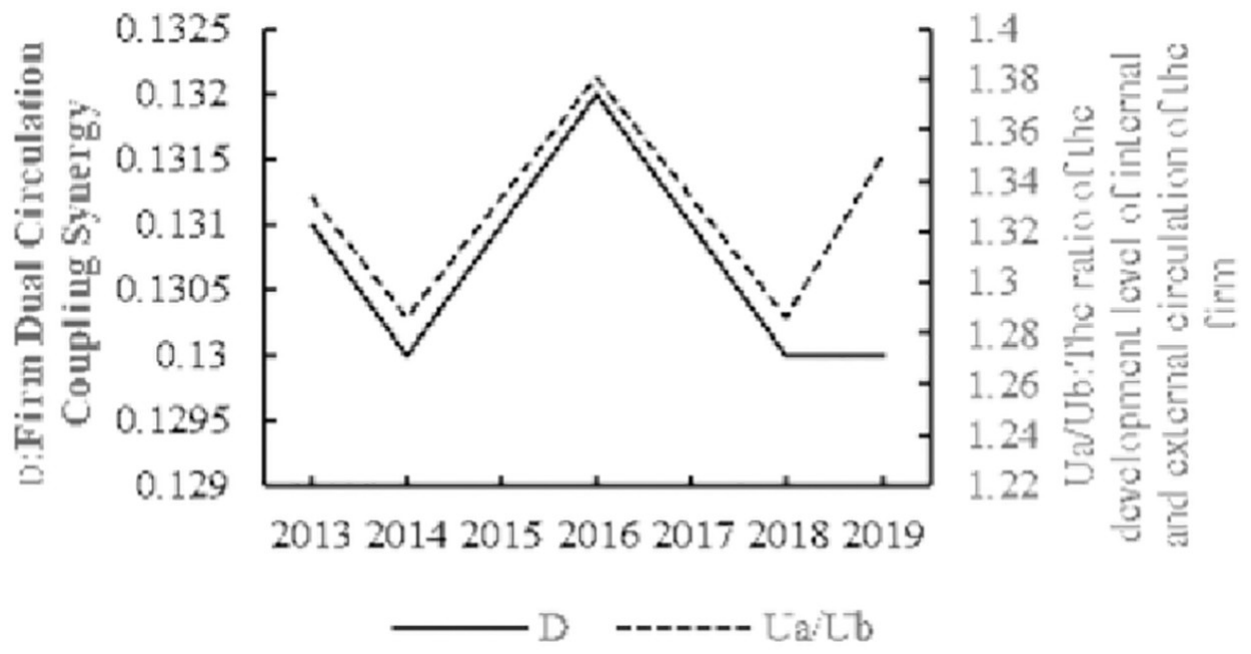
Coupling synergy. D represents the degree of the firm’s dual circulation coupling synergy. Ua/Ub represents the ratio of the average development level of the internal circulation system to the average development level of the external circulation system. If the ratio is greater than 1, the internal circulation system is ahead of the external circulation system. It is clear that the firm dual circulation coupling synergy is at a low-level, and the development of the inner cycle system of firms is ahead of the outer cycle system.

In Fig 2, the Ua/Ub represents the ratio of the average development level of the internal circulation system to the average development level of the external circulation system. The ratio is then used to measure the leading or lagging degree of the internal circulation system relative to the external circulation system. If the ratio is greater than 1, the internal circulation system is ahead of the external circulation system; if the ratio is less than 1, the internal circulation system is lagging behind the external circulation system; if the ratio is equal to 1, the two are developing simultaneously. Fig 2 shows that the firm dual circulation coupling synergy is at a low-level, which indicates that there is a coupling synergy relationship between the firm’s internal and external circulation systems. But the degree of coupling synergy is lower, this is consistent with Du et al. (2023) [38]finding that firms’ overall level of "domestic sales-export" coupling synergy is low. The degree of coupling synergy is lower because Chinese firms have long been overly dependent on foreign trade, foreign capital and foreign technology. This has led to a number of problems: suppression of China’s domestic market demand; lack of independent innovation capability of local processing trade firms; restriction of key core technologies; extreme vulnerability of supply chains; intensification of overcapacity in some entities; slow capital turnover; lack of willingness to invest. These disadvantages have subsequently hindered the development of Chinese firms, resulting in poor internal and external circulation, and ultimately leading to poor coupling synergy between the internal and external circulation of firms.

The development of the inner cycle system of firms is ahead of the outer cycle system, which is due to locking the low end of the GVC in the process of integrating Chinese firms into the GVC and becoming a global factory. Study shows the quality of the outer cycle development of firms is low, while the inner cycle development shows great development potential, which is consistent with the conclusion of Zhao et al. (2022) [12]. The existence of unbalanced development between the internal and external circulation system makes it impossible to improve the level of coupling synergy. Further, the use of internal circulation to drive the development of external circulation and thus improve the degree of coupling synergy becomes the key to firm development.

**Fig 3:**
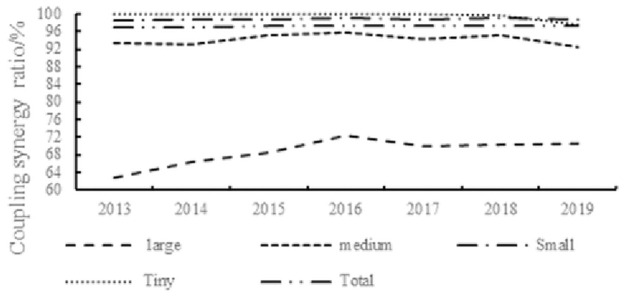
Percentage of low coupling synergy by size. The lines show the percentage of firms of different sizes that are in the low coupling synergy stage between 2013 and 2019. It is clear that the dual circulation coupling synergy degree of large firms is better than that of other firms.

With reference to the "*Statistical Classification of Large*, *Small*, *Medium and Micro Firms (2017)*" issued by the National Bureau of Statistics, the sample is divided into large firms and small, medium and micro firms based on the number of employees and the business revenue of the firms in the year. As seen from Fig 3, the dual circulation coupling synergy degree of large firms is greater than that of other sizes. The percentage of large firms in the range of low coupling synergy is 62%-73%, while the percentage of other firms in the range of low coupling synergy is over 90%. The intuitive explanation is that large firms are more connected to both domestic and international markets as they have better human resources, sufficient capital, domestic and foreign trade experience, and the ability to innovate, and are more likely to occupy a supply chain master position. Subsequently, in the longer term, the government should focus more on the interplay of dual circulation in small and medium-sized firms, particularly the impact of external uncertainties. Some firms have “unusual” difficulties in turning foreign sales into domestic sales. Consequently, the internal and external cycle system, which is not yet able to interact on a higher level, should be used to design strategic methods to facilitate growth in firms of different sizes, and thus avoid a one-size-fits-all approach.

Table 3 shows that 99% of the firms’ dual circulation subsystems are in the low coupling synergy stage. During the sample period, the proportion of firms in the low coupling synergy stage between the external and internal circulation innovation system and the internal and external circulation human capital system fluctuates and decreases. This indicates an increase in the coupling synergy of these two systems. In other words, the innovation element of the internal circulation and the human capital element of the external circulation are the most important elements in the process of mutual coordination and promotion of the development of the internal and external circulation of firms. This precisely reflects China’s current development concept of an innovation-driven and talent-strong country. Overall, the degree of coupling synergy is low because China’s key technologies are blocked by the western developed countries, and the trade war with the USA forms international barriers, leading to poor internal and external firm cycles. Therefore, firms should strengthen the capacity for independent innovation, and take advantage of domestic and international markets, which will also help firm dual circulation reach a high level of coupling synergy.

**Table 3:** The proportion of synergy between each subsystem of the internal and external cycle of the firm and the internal and external cycle coupling respectively. Unit: %.

year	External Circulation				Internal circulation			
	Ind-inno	Hum-cap	Ind-ac-re	Funds-liq	Tech-inno	Hum-cap	Ind-ac-re	Funds-liq
**2013**	99.32	99.60	99.73	99.84	100	99.88	100	100
**2014**	99.06	99.41	99.97	99.85	100	99.82	100	100
**2015**	99.43	99.50	99.92	99.82	100	99.79	100	100
**2016**	99.03	99.52	99.92	99.80	100	99.61	100	100
**2017**	99.69	99.64	99.78	99.92	100	99.72	100	100
**2018**	99.46	99.62	99.65	99.97	100	99.67	100	100
**2019**	99.01	99.72	99.76	99.92	100	99.56	100	100

## Survival model construction and variable selection

The purpose of this study is to explore the impact of a firm’s dual circulation coupling synergy on firm survival. According to Fernandes and Paunov (2015) [49], the firm *i* survival event is defined by firm survival time *T*. If firm *i* exists in year *t* and dies in year *t* + 1 (*c*_*i*_ = 1), firm *i* exits. If right-hand side data censoring or firm does not die (*c*_*i*_ = 0), firm *i* survives, and the survival model can handle the right-hand censoring problem well, and for the data existence of the left censoring problem, we deals with it according to Baoqun et al. (2015) [50]. The survival function describes the probability that the firm will persist in the sample for more than *t* years, and is defined as follows:

$$S\left(t_{i}\right)=\text{Pr}\left(T_{i}>t\right)=\prod_{k=1}^{t}\left(1-h_{ik}\right)$$ (9)

Where $T_{i}=\text{min}\left\{T_{i}^{*},C_{i}^{*}\right\},T_{i}^{*}$ is the determined survival time, and $C_{i}^{*}$ is the survival time in case of deletion. The firm risk function is defined as the probability that the firm will die at year *t* and survive at year *t* − 1:

$$h_{i}\left(t\right)=\text{Pr}\left(t-1<T_{j}<t|T_{j}>t-1\right)=\text{Pr}\left(t-1<T_{j}\leq t\right)/\text{Pr}(T_{j}>t-1)$$ (10)

Kaplan-Meier’s nonparametric estimation (Kaplan and Meier, 1958) [51] is able to use the full information of right-censored and non-censored data and is stable to right-censoring; therefore, K-M is used for the estimation of the survival function, which is estimated as:

Sti=∏k=1t(nk−dk)nk (11)

*n*_*k*_ denotes the number of firms at risk of exit in period *k*.*d*_*k*_ denotes the number of actual exits observed in period *k*. The risk function is:

hit=dknk (12)

To obtain valid estimates, a regression model is used to investigate the causal relationship between the risk probability *h*_*ik*_ and the explanatory variables, as well as the inclusion of control variables that affect the firm’s survival. The discrete-time Cloglog model is constructed as follows:

$$cloglog\left(1-h_{it}\right)=\text{log}\left(-\;\text{log}\left(1-h_{it}\right)\right)=\alpha_{0}+\beta_{1}Coupling_{it}+\beta_{2}Control_{it}+\tau_{t}+\gamma_{t}+u_{k}+\varepsilon_{itk}$$ (13)

*h*_*it*_ indicates the discrete-time risk rate, and the larger the explanatory variable *cloglog*(1 − *h*_*it*_), the higher the risk rate or lower the survival probability of the firm. *Coupling*_*it*_ is the explanatory variable, firm dual circulation coupling synergy index. *β*_1_ is the parameter to be estimated, indicating the actual impact of the firm dual circulation coupling synergy on the survival of the firm. *β*_1_ < 0 indicates that in respect of the firm dual circulation, the higher the coupling synergy, the lower the exit risk of the firm. *Control*_*it*_ are control variables, including capital intensity, firm age and age’s secondary term, firm size, government subsidies, profitability and financing constraints. *τ*_*t*_ is the benchmark risk rate. *γ*_*t*_ is the time fixed effect. *u*_*k*_ is the industry fixed effect. *ε*_*itk*_ is random disturbance term.

Control variables. Firms with high capital intensity are conducive to enhancing their production and financing capacity, thus improving their probability of survival; this is measured by the ratio of fixed assets to employees. Newly established firms face a greater risk of failure, while those with increased age are better able to gradually adapt to the external environment, and improve their probability of survival. However, some scholars argue that there is a non-linear relationship between firm age and firm survival (Yu et al., 2015) [52] and that as firms age, they may exhibit relatively rigid behavior and fail to adapt quickly to market competition, limiting their chances of survival. Therefore, we introduce two indicators: firm age and its quadratic term, which are measured by subtracting the year of firm establishment from the year of the sample observation period and then taking the logarithm; the closer the firm size is to the efficient size of the market, the lower the probability of firm death (Audretsch et al., 1994) [53], as is measured by the logarithm of the number of employees in the firm. Profitability is the fundamental motivation for firms to enter and exit the market, and so improving profitability can help reduce exit risk and thus prolong firm survival, as measured by the ratio of operating profit to operating revenue. The lower the financing constraint to which a firm is subjected, the higher the probability of survival, as measured by the SA index(SA = -0.737×Size+0.043×Size2–0.040×Age) (Liu et al., 2021) [54]. Government subsidies send positive signals to internal and external stakeholders, which helps reduce a firm’s survival risk and increase its confidence in continuing to operate, as measured by the ratio of government subsidy revenue to the firm’s measured as a proportion of total assets. Finally, the industry and year are fixed.

The descriptive statistical analysis of the main variables is shown in Table 4. In the sample period, the mean values of indicators are higher for non-exiting firms than for exiting firms. This shows that the dual circulation coupling synergy of firms and control variables make it more likely for firms to stay in business.

**Table 4:** Definitions and descriptive statistics of the variables used in the empirical analysis.

Variables	Variable Definition	Means		Std		Min		Max	
**d**	Exit = 1, No exit = 0	0	1	0	1	0	1	0	1
**Coupling**	Firm dual circulation coupling synergy	0.132	0.11	0.057	0.031	0.1	0.1	0.426	0.425
**lnage**	Firm age	2.283	2.232	0.574	0.579	0.693	0.693	3.367	3.367
**lnage2**	Firm age square	4.335	4.221	1.286	1.306	0.693	0.693	6.666	6.665
**lnscale**	Firm size	3.507	2.265	1.391	1.176	0.693	0.693	7.325	7.325
**CapitalIntensity**	Capital intensity	2.951	2.716	1.781	1.953	0	0	7.787	7.787
**Profit**	Profit level	-0.072	-0.145	0.432	0.506	-2.978	-2.977	0.556	0.555
**Subsidies**	Government subsidies	0.007	0.003	0.021	0.015	0	0	0.142	0.141
**SA**	Financing constraints	-3.284	-3.38	0.412	0.344	-4.013	-4.013	-1.518	-1.518
**N**	Observations	56779	8346	56779	8346	56779	8346	56779	8346

## Empirical results and analysis

### Business survival K-M (Kaplan-Meier) estimates

Firstly, we analyze the impact of firm dual circulation coupling synergy on corporate survival. According to the estimated survival function of the entire sample, the survival rate of firms gradually declines with time. 94.78% of firms survive for more than one year, 5.22% withdraw one year after the start of the sample period, 84.89% survive for more than five years, 15.11% of firms exit the market. The survival rate of firms in ZGC is higher than that of firms in traditional industries (60.24%), but it is still on a downward trend. Thus, it is necessary to consider how to improve the survival rate of firms.

#### Table 5. Estimated firm survival function

Survival time of 2 years was used as an example. During the observation period 54264 firms survived longer than 2 years.2759 firms exited the market. 5393 firms were lost (censored). The firm survival rate is 89.96%. As a result 46112(54264-2759-5393 = 46112.) firms have survived longer than 3 years.

**Table 5:** Estimated firm survival function.

Survival time	N	exiting	lost	Survival rate	SD	95% confidence interval	
1	65125	3401	7460	0.9478	0.0009	0.9460	0.9495
2	54264	2759	5393	0.8996	0.0012	0.8972	0.9019
3	46112	659	8486	0.8867	0.0013	0.8842	0.8893
4	36967	1019	3205	0.8623	0.0015	0.8594	0.8651
5	32743	508	9347	0.8489	0.0016	0.8458	0.8519
6	22888						

Note: The firm survival results in year 6 are beyond the sample observation period, so the survival status is missing, as below.

To investigate the impact of firm dual circulation coupling synergy on firm survival, we estimate and compare the survival functions of firms with high and low coupling synergy using firm dual circulation coupling synergy as a stratification variable; the estimation results are provided in Table 6. Survival times differ dramatically with different degrees of dual circulation coupling synergy. Firms with strong dual circulation coupling synergy survive substantially longer than those with low dual circulation coupling synergy. In terms of survival rate, firms with high dual circulation coupling synergy have a 98.19% chance of surviving for more than one year and a 96.46% chance of surviving for more than five years, while firms with a low dual circulation coupling synergy have a 94.68% chance of surviving for more than one year and an 84.53% chance of surviving for more than five years. In terms of survival rate, firms with strong dual circulation coupling synergy outperform those with low dual circulation coupling synergy. In terms of average survival time, firms with high dual circulation coupling synergy have a survival time of 4.79 years, while firms with low dual circulation coupling synergy have a survival time of 3.94 years, indicating that the degree of dual circulation coupling synergy can extend firm survival time by 0.85 years. This demonstrates that differences in firm longevity can be caused by differences in the degree of dual circulation coupling synergy between firms.

**Table 6:** Estimated survival function of observed firms.

Survival time	N	exiting	lost	Survival rate	SD	95% confidence interval	
**Coupling_0 = 0: Low Coupling Synergy**				**Average business survival time: 3.94**			
1	63299	3368	7207	0.9468	0.0009	0.9450	0.9485
2	52724	2747	5354	0.8975	0.0012	0.8950	0.8999
3	44623	654	8371	0.8843	0.0013	0.8817	0.8869
4	35598	1016	3189	0.8591	0.0015	0.8561	0.8620
5	31393	502	9182	0.8453	0.0016	0.8422	0.8485
6	21709						
**Coupling_0 = 1: High coupling synergy**				**Average business survival time:4.79**			
1	1826	33	253	0.9819	0.0031	0.9747	0.9871
2	1540	12	39	0.9743	0.0038	0.9657	0.9807
3	1489	5	115	0.9710	0.0041	0.9619	0.9780
4	1369	3	16	0.9689	0.0042	0.9594	0.9762
5	1350	6	165	0.9646	0.0046	0.9544	0.9725
6	1179						

**Fig 4:**
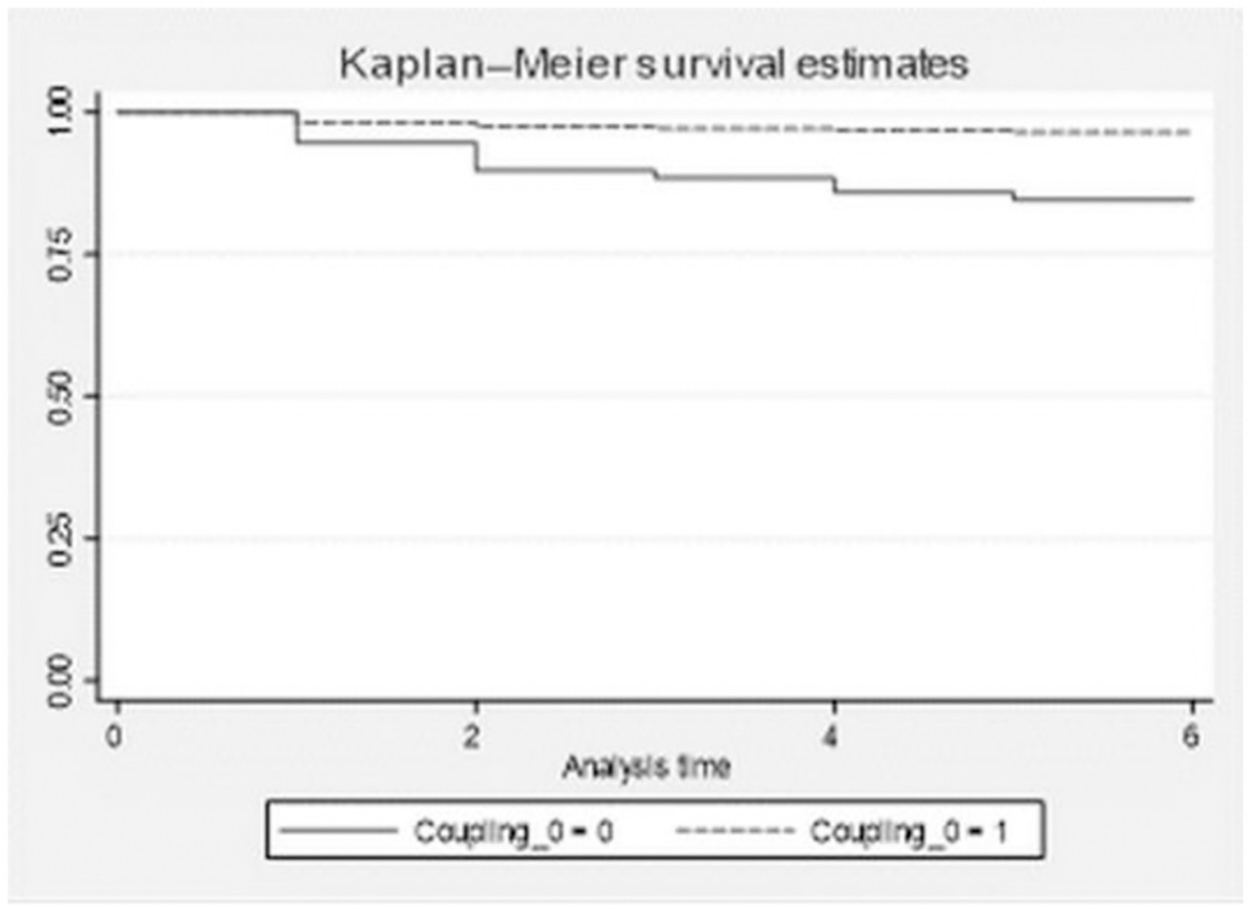
K-M survival curve. The solid line represents the K-M estimate for the low coupling synergy firm, and the dotted line represents the K-M estimate for the high coupling synergy firm. The survival curves of high dual circulation coupling synergy firms are higher than those of low dual circulation coupling synergy firms.

The Kaplan-Meier survival estimation curve, as shown in Fig 4 is more intuitive than Table 6. The survival curves of high dual circulation coupling synergy firms are higher than those of low dual circulation coupling synergy firms.

**Fig 5:**
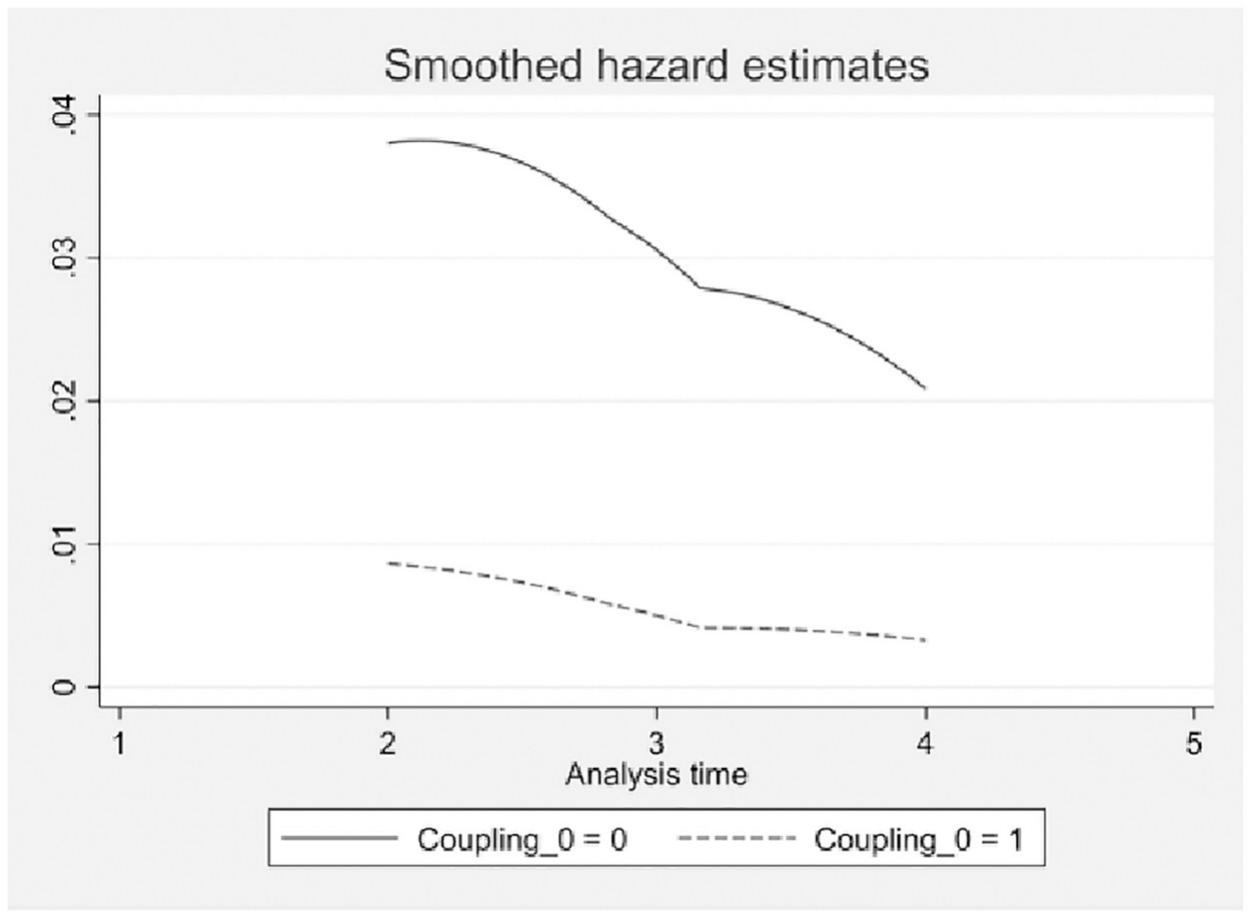
Risk curve. The solid line represents the risk estimate for the low coupling synergy firm, the dotted lines represent the risk estimate for the high coupling synergy firm. The survival risk curve of firms with high dual circulation coupling synergy is lower than that of firms with low dual circulation coupling synergy.

According to the risk estimation graph in Fig 5, the survival risk curve of firms with high dual circulation coupling synergy is lower than that of firms with low dual circulation coupling synergy; this implies that firms with greater dual circulation coupling synergy have a lower survival risk.

Based on the above preliminary analysis, it can be found that the impact of the degree of dual circulation coupling synergy on firm survival is significantly different, and firms with a high degree of dual circulation coupling synergy will prolong their survival time and reduce their exit risk. However, the K-M estimation only depicts the impact of the degree of dual circulation coupling synergy on the survival probability of a firm and does not reflect that the occurrence of an “exit” event is influenced by a firm’s own factors. Therefore, it is necessary to conduct a more rigorous empirical test using a discrete-time Cloglog survival model.

### Cloglog survival model baseline regression

Table 7 reports the results of the discrete-time Cloglog survival model estimation. Columns (1) to (5) are estimates of the baseline model Eq (5). The estimated coefficient sign and significance level of the core explanatory variable do not change substantially, indicating that the regression results are relatively robust. The following analysis is based on the regression results in Column (5): the estimated coefficient of the core explanatory variable is negative and significant at the 1% level of significance, which indicates that firm dual circulation coupling synergy reduces the risk rate of a firm exiting from the market, and so can prolong the survival duration of the firm. The conclusion is similar to Du et al. (2023) [38] and Chen et al. (2012) [22]. Hypothesis 1 is verified.

**Table 7:** Baseline estimation of the cloglog model.

Variables	(1)	(2)	(3)	(4)	(5)
	Cloglog	Cloglog	Cloglog	Cloglog	Cloglog
**Coupling**	-19.591***	-1.390**	-2.325***	-1.423**	-2.209***
	(-19.40)	(-2.41)	(-3.84)	(-2.44)	(-3.62)
**lnage**		2.602***	8.165***	2.828***	7.968***
		(3.20)	(10.06)	(3.45)	(9.71)
**lnage** ^**2**^		-1.075***	-3.562***	-1.208***	-3.492***
		(-2.97)	(-9.89)	(-3.31)	(-9.58)
**lnscale**		-0.708***	-0.712***	-0.718***	-0.720***
		(-62.26)	(-61.62)	(-62.54)	(-61.68)
**CapitalIntensity**		-0.021***	-0.017***	-0.023***	-0.019***
		(-3.57)	(-2.70)	(-3.78)	(-2.89)
**Profit**		-0.174***	-0.235***	-0.186***	-0.243***
		(-7.91)	(-10.20)	(-8.39)	(-10.50)
**Subsidies**		-10.318***	-4.444***	-9.975***	-4.150***
		(-9.83)	(-5.25)	(-9.56)	(-4.94)
**SA**		0.044	0.195***	0.019	0.162***
		(0.95)	(4.15)	(0.39)	(3.40)
**Constant**	0.305***	-0.878***	-1.677***	-0.945	-2.075**
	(2.73)	(-2.87)	(-5.48)	(-0.94)	(-2.00)
**industry**	No	No	No	Yes	Yes
**year**	No	No	Yes	No	Yes
***N***	65125	65125	54073	65058	54023
**Log likelihood**	-23870.384	-21531.911	-19276.929	-21361.583	-19163.032

*Note*:*t* statistics in parentheses

* *p* < 0.1,

** *p* < 0.05,

*** *p* < 0.01.

### Robustness tests

#### Sub-sample regression

Different models were used to test the robustness of the above regression results. Columns (1) to (5) in Table 8 show the regression results of different models; the results are robust. Next to be considered are the firms more newly established after 2013 (the beginning of the sample). The advantage of the new firms is that they enter the market during the sample period, allowing us to observe more precisely the behavior of firms entering and exiting the market. Column (6) shows the estimated results; the estimated coefficient was -2.801, which was significant at the 10% level. The baseline regression results are robust.

**Table 8:** Robustness test results.

Variables	(1)	(2)	(3)	(4)	(5)	(6)
	Weibull	Exponential	Gompertz	ATF	logit	Cloglog
**Coupling**	-2.666***	-2.465***	-2.551***	0.235	-1.885***	-2.801*
	(-5.18)	(-4.84)	(-4.98)	(0.99)	(-2.92)	(-1.86)
**Constant**	-2.479**	-2.478**	-2.509**	2.224***	-2.262*	1.851**
	(-2.38)	(-2.37)	(-2.40)	(3.00)	(-1.84)	(2.01)
**Control**	Yes	Yes	Yes	Yes	Yes	Yes
**industry**	Yes	Yes	Yes	Yes	Yes	Yes
**year**	Yes	Yes	Yes	Yes	Yes	Yes
**N**	65125	65125	65125	65125	54023	7015
**Log likelihood**	-23511.315	-24506.664	-24356.374	-23204.691	-19151.324	-2801.076

#### Endogeneity test

In order to overcome the possible estimation bias caused by the endogeneity problem of the core explanatory variable of the dual circulation synergy of firms, the instrumental variables approach is applied. Fisman and Svensson (2007) [55] use industry-region averages of property rights as an instrumental variable for firm property rights in their study of property rights protection and economic growth. They argue that if the endogeneity problem only exists at the firm level (and not at the industry or regional level), then removing industry- and region-specific property rights components yields property rights factors that only affect the growth of individual firms. Firms in the same size-age-industry face similar external environments and firm characteristics. Therefore, firm dual circulation coupling synergy has some relevance, and should not have a direct impact on the survival of other firms. This paper employs Fisman and Svensson’s (2007) method. The firm size-firm age-industry mean value of firm dual circulation coupling synergy serves as the corresponding instrumental variable (Li et al., 2010; Kang, 2013; Huang et al., 2020) [56–58]. The estimation follows the usual two-step estimation method: (1) estimation of the firm dual circulation coupling synergy using the firm size-firm age-industry mean, which yields the fitted values; (2) re-estimation of the fitted values using the instrumental variables method by applying them to the Cloglog survival model. Table 9 shows that the estimation results remain robust.

**Table 9:** Results of endogeneity test.

Variables	(1)	(2)
	Cloglog	Logit
**IV Coupling**	-1.986***	-1.499**
	(-3.37)	(-2.05)
**Constant**	-2.098**	-2.308*
	(-1.98)	(-1.88)
**Control**	Yes	Yes
**industry**	Yes	Yes
**year**	Yes	Yes
***N***	54023	54023
**Log likelihood**	-19167.22	-19154.78

#### Generalized propensity score matching model

**Fig 6:**
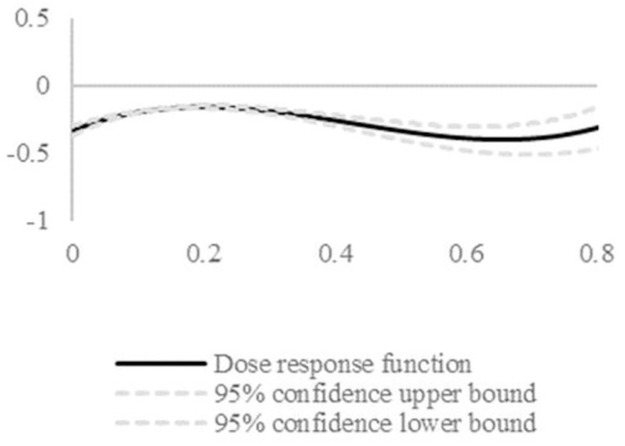
Dose response function. Solid lines represent the dose response function, whilst the dotted line represents the 95% confidence interval. The relationship between the degree of coupling synergy of firm dual circulation and firm survival is below 0, which indicates that the firm survival risk keeps decreasing as the degree of coupling synergy of firm dual circulation increases.

To avoid endogeneity problems caused by sample self-selection bias, this paper proposes to use the generalized propensity score matching model (GPSM) to conduct a “counterfactual” analysis to overcome the sample self-selection bias. The traditional propensity score matching model (PSM) can only be used to test the treatment effect of treatment variables of type 0–1. According to existing research, GPSM can handle multivariate or continuous treatment variables and is appropriate for assessing the effect of dual circulation coupling synergy and firm survival. Fig 6 depicts the robustness test results.

**Fig 7:**
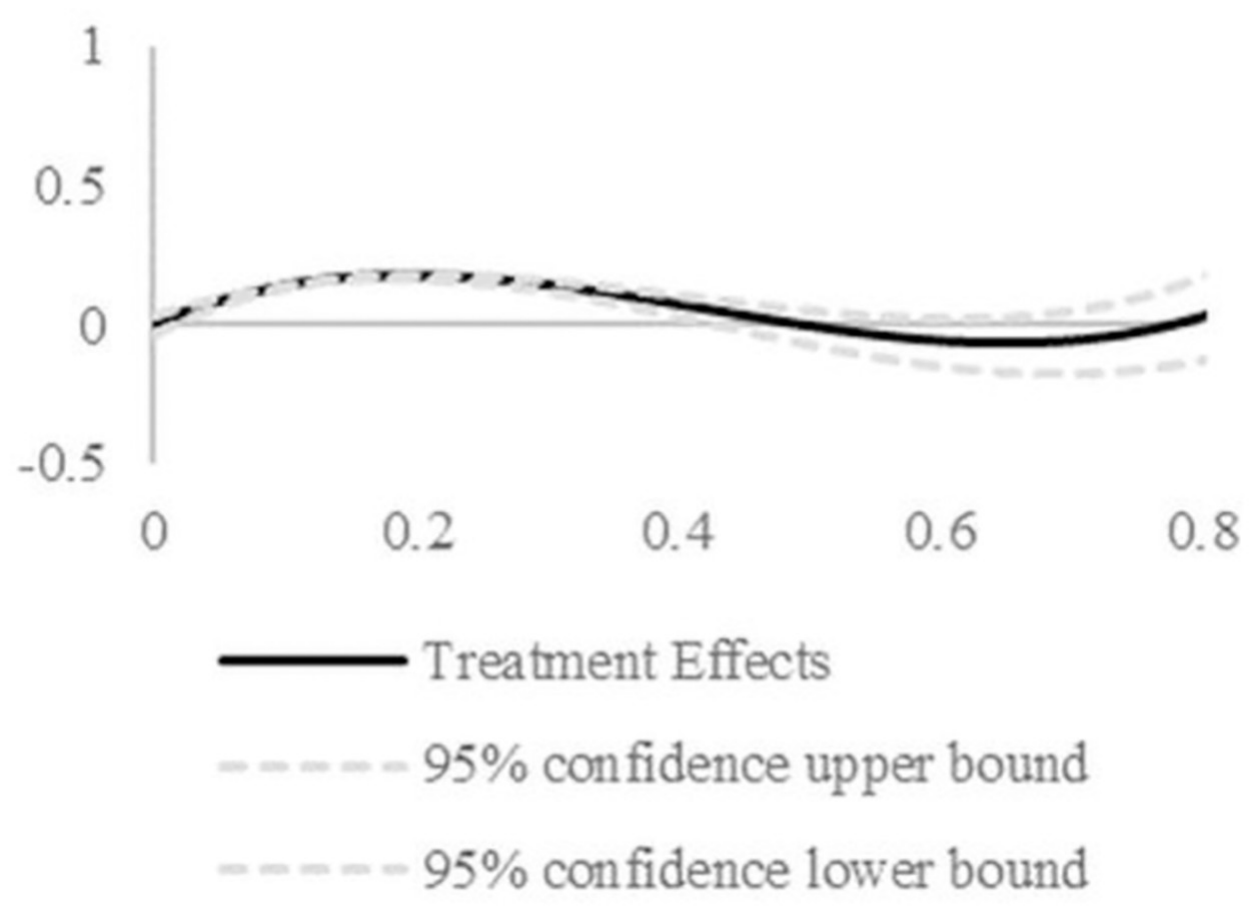
Treatment effect function. Solid lines represent treatment effect function whilst the dotted line represents the 95% confidence interval. This indicates that the firm survival risk continuously decreases in line with the increasing intensity of the firm’s dual circulation coupling synergy.

The relationship between firm dual circulation coupling synergy and firm survival risk is obtained using the GPSM matching method as shown in Figs 6 and 7. Fig 6 reports the average dose-response function plot. Fig 7 reports the effect of different degrees of coupling synergy on firm survival risk (treatment effect). In Fig 6, the relationship between the degree of coupling synergy of firm dual circulation and firm survival is below 0 and shows a more obvious decreasing trend from 0.2. This indicates that the firm survival risk continues to decrease as the degree of coupling synergy of firm dual circulation increases. The treatment effect of firm dual circulation coupling synergy strength on firm survival risk is also calculated by considering the difference between firm survival risk under different firm dual circulation coupling synergy strengths and firm survival risk under 0 strength. The results are shown in Fig 7. It can also be seen that firm survival risk decreases with an increase in the strength of firm dual circulation coupling synergy, which shows that the effect of firm dual circulation coupling synergy on firm survival is robust.

### Mechanism analysis

We choose market demand effect, innovation effect, and resource allocation effect as mediating variables. Firm sales revenue can better reflect the market demand capability of the product, and the ratio of firm sales revenue to total industrial output is chosen to measure market demand (*Demand*). Innovation effect (*Innovation*) is measured by the proportion of new product output value to total industrial output value of the firm. Resource allocation efficiency is measured by total factor productivity (*TFP*), Measured by OP method. Existing studies fail to fully consider human capital-related factors when measuring resource allocation efficiency. Therefore, this paper considers the allocation efficiency of labor capital (*Labor*) at the level of educational attainment (Qu, 2020) [59], which is measured by using the ratio of the number of employees’ masters and doctoral degrees to owner’s equity. We use the mechanism test method of Li et al. (2021) [60] to test the influence mechanism. The model is established as follows:

$$Demand=\varphi_{0}+\varphi_{1}Coupling_{it}+\varphi_{2}control_{it}+\lambda_{0i}+\theta_{0t}+\varepsilon_{0it}$$ (14)

$$Innovation=\gamma_{0}+\gamma_{1}Coupling_{it}+\gamma_{2}control_{it}+\lambda_{1i}+\theta_{1t}+\varepsilon_{1it}$$ (15)

$$TFP=\omega_{0}+\omega_{1}Coupling_{it}+\omega_{2}control_{it}+\lambda_{3i}+\theta_{3t}+\varepsilon_{3it}$$ (16)

$$Labor=\rho_{0}+\rho_{1}Coupling_{it}+\rho_{2}control_{it}+\lambda_{4i}+\theta_{4t}+\varepsilon_{4it}$$ (17)

In Eqs (14)–(17), *φ*_1_, *γ*_1_, *ω*_1_, *ρ*_1_ denote the degree of influence of firm dual circulation coupling synergy on the three mechanism variables.

The results are shown in Table 10. Column (1) shows that the estimated coefficient of the effect of firm dual circulation coupling synergy on market demand is significantly positive at the 5% level, for every 1% increase in firm dual circulation coupling synergy, the firm market demand space expands by 8.5%. Firm dual circulation coupling synergy expands the firm market space and improves survivability. Hypothesis 2 is verified. Column (2) shows that the estimated coefficient of firm dual circulation coupling synergy on firm innovation is significantly positive at the 1% level, which indicates that it promotes firm innovation, and for every 1% increase in firm dual circulation coupling synergy, firm innovation capability increases by 10.8%. Firm dual circulation coupling synergy promotes innovation and improves survivability. Hypothesis 3 is verified. Column (3) and (4) show that the estimated coefficients of firm dual circulation coupling synergy on TFP and labor resource allocation are significantly positive at the 10% and 5% levels, respectively, indicating that firm dual circulation coupling synergy can optimize resource allocation. For every 1% increase in firm dual circulation coupling synergy, TFP increases by 84.4% and labor resource allocation efficiency increases by 0.1%. Firm dual circulation coupling synergy enhances resource allocation efficiency and improves survivability. Hypothesis 4 is verified.

**Table 10:** Intermediate mechanism test.

Variables	(1)	(2)	(3)	(4)
	Market demand effect	Innovation effect	Resource allocation effect	
	Demand	Innovation	TFP	Labor
**Coupling**	0.085**	0.108***	0.844*	0.001**
	(2.51)	(3.89)	(1.89)	(2.02)
**Constant**	0.045	-0.060	7.466***	-0.002
	(0.69)	(-1.26)	(7.02)	(-1.64)
**Control**	Yes	Yes	Yes	Yes
**Individual**	Yes	Yes	Yes	Yes
**year**	Yes	Yes	Yes	Yes
**N**	65125	65125	65125	65125
**adj. R** ^**2**^	0.004	0.008	0.005	0.008

### Further analysis

Baseline regression analyze the impact of firm dual circulation coupling synergy on firm survival, but they are based on a full sample analysis and do not consider the presence of firm heterogeneity. Therefore, the next section considers how firm dual circulation coupling synergy affects the survival of different types of firms.

#### Firm life cycle

As a start-up firm has just entered the market, its advantages in core technology, financing, talent absorption as well as internal and external collaboration in the market are weak, the role of firm dual circulation coupling synergy in promoting the survival of the start-up firm is not obvious. As firms transition from the start-up stage to the growth stage, they continuously optimize the elements and platforms for internal and external technology innovation, invest more in R&D, and attract more excellent talents. In the process, firms continue to integrate with the internal and external markets. Therefore, their ability to use dual circulation and convert to the market is continuously improved. Therefore, the role of dual circulation coupling synergy in promoting firm survival becomes more obvious during the growth period. After entering the maturity period, firm advantages are further improved, with their market share and market demand space continuing to expand. Therefore, firm dual circulation coupling synergy is more conducive to survival in the maturity period; once firms enter the recession period, the advantages decline, market demand space shrinks, innovation ability decreases, resource allocation efficiency decreases, and hence the promotion effect is reduced. Table 11 shows that the effect of firm dual circulation coupling synergy on the survival risk of startup and recessionary firms is not significant, but has a significant effect on the stages of growth and maturity.

**Table 11:** Firm life cycle and survival time heterogeneity.

Variables	Life cycle				Survival time	
	Start-up	Growing-up	Mature	Recession	Newly	Non-Newly
**Coupling**	-1.388	-2.232*	-3.813***	-1.562	-1.959	-2.347***
	(1.377)	(1.272)	(1.268)	(1.040)	(1.263)	(0.698)
**Constant**	-2.554**	-0.685	-2.301**	-0.239	-11.44***	-1.133
	(1.167)	(0.891)	(1.059)	(0.649)	(2.162)	(1.182)
**Control**	Yes	Yes	Yes	Yes	Yes	Yes
**industry**	Yes	Yes	Yes	Yes	Yes	Yes
**year**	Yes	Yes	Yes	Yes	Yes	Yes
**N**	9245	14154	10625	19811	8702	45273

#### Firm survival time

There are significant differences in the viability of firms with different survival times, with those newly established being relatively less viable and more sensitive to changes in the external environment (Bian et al., 2021) [33]. Newly established firms obtain more government support than established firms, but they are limited with their own resources, take up a relatively small market space and have weak competitiveness. Consequently, the effect of firm dual circulation coupling synergy on their survival risk is insignificant. The more established firms have a more stable market space and better resources, and so firm dual circulation coupling synergy can promote the expansion of market demand space, improve innovation capacity and further optimize resource allocation, thus reducing the survival risk. Newly-established firms are considered to be those established less than five years ago; non-newly established firms are considered to be those established over five years ago. Table 11 shows that the effect of firm dual circulation coupling synergy on survival risk is negative for both newly and non-newly established firms. However, whilst insignificant for the former, firm dual circulation coupling synergy effect increases the survival rate for the latter.

#### Firm size

Large firms usually occupy greater market demand space and resources. However, they are often slow to adjust themselves in the face of adverse external environmental changes because of their large scale. This leads to squeezed market demand space and less efficient resource allocation, which results in firm dual circulation coupling synergy having a less obvious impact on their survival risk. Medium, small and micro firms have the characteristic of “small boat is good to turn around”. Although it scale is not as large as that of large firms, the market demand space is relatively small. However, they can quickly adapt to changes in the external environment, and their strategy adjustment is more flexible, which is especially true for export-oriented firms; in the face of weakening demand in foreign markets, they can quickly shift their focus to the domestic market, and achieve a state of dual circulation coupling synergy in a relatively short period of time and ensure their own survival. Table 12 shows that the effect of firm dual circulation coupling synergy on the survival risk of medium, small and extra small firms is significantly negative, while for large firms it is positive but not significant. This may be due to large firms being relatively stable and difficult to break through in terms of market demand, innovation and resource allocation, so the contribution of firm dual circulation coupling synergy on their survival risk is offset.

**Table 12:** Firm size and firm ownership heterogeneity.

Variables	Firm Ownership			Size			
	Private	State-owned	Others	Large	Medium	Small	Micro
**Coupling**	-4.116***	-1.171	-1.857	1.666	-4.608**	-5.041***	-4.257***
	(0.863)	(1.461)	(1.322)	(1.310)	(1.984)	(1.076)	(1.234)
**Constant**	-1.165	-0.153	1.184	-6.808**	-6.454***	-1.739**	-0.725
	(0.741)	(1.307)	(1.554)	(2.846)	(1.812)	(0.803)	(1.083)
**Control**	Yes	Yes	Yes	Yes	Yes	Yes	Yes
**industry**	Yes	Yes	Yes	Yes	Yes	Yes	Yes
**year**	Yes	Yes	Yes	Yes	Yes	Yes	Yes
**N**	45320	5067	3532	2186	6822	28773	15563

Note: Others include Hong Kong, Macau, Taiwan and foreign firms.

#### Nature of firm ownership

As an important carrier of the government’s macro-control functions, state-owned firms bear the policy burden of improving employment, maintaining economic stability and increasing welfare, hence their operational efficiency and market competitiveness are relatively low (Wu, 2012) [61]. Therefore, firm dual circulation coupling synergy can expand their market demand space, enhance innovation capacity and resource allocation efficiency, and thus promote their survival. For private firms, dual circulation coupling synergy increases their market space, and in the face of external uncertainties, they can flexibly adjust according to changes in market demand, which makes it easier for them to buffer the impact of market changes. As private firms are the driving force behind innovation, they are more likely to easily integrate into domestic or foreign markets, connect two markets, adjust production efficiency, realize dual circulation coupling synergy, engage in more efficient production activities, and extend their survival time. For others, dual circulation coupling synergy can expand their market demand space, enhance the efficiency of resource allocation, and have a catalytic effect on survival. Table 12 shows that the effect of firm dual circulation coupling synergy on the survival risk of firms with different ownership properties is negative, but only the effect of private firms is significant. This indicates that firm dual circulation coupling synergy further enhances the innovation capability and resource allocation efficiency of private firms and expands their market space.

## Discussion

In this paper, we focus on the impact of firm dual circulation coupling synergy on firm survival rates. Similar to previous studies of firm survival [22, 24], firms can increase their probability of survival by utilizing both markets. However, we find that firm dual circulation coupling synergy enhances firm survival. One possible explanation is that the dual circulation coupling synergy allows the firm’s risks to be spread between the two markets, thus strengthening the firm’s own risk resistance, which assists a firm’s survival. Furthermore, firm dual circulation coupling synergy expands the firm market space, promotes innovation, enhances resource allocation efficiency, and improves survivability.

The findings of this paper are important for the development of future relevant policies. However, as a firm’s dual circulation relationship is very complex, there are some shortcomings and areas that require further improvement and supplementation. Due to data limitations, this study does not discuss whether there is a difference in firm survival rates by firm dual circulation coupling synergy in different regions at the spatial level; this is a potential area of focus for future studies.

## Conclusions

The main findings of the paper are as follows. First, dual circulation coupling synergy can increase a firm’s survival rate, and the greater the degree of coupling synergy, the more visible the promotion effect. Second, firm dual circulation coupling synergy primarily improves market demand, innovation, and resource allocation effects, and then enhances firm survival. Third, dual circulation coupling synergy has a facilitating effect on the survival of growth and maturity firms, medium, small, and micro firms, private firms, and non-newly established firms. It does not contribute significantly to the survival of other types of firms.

The recommendations are as follows: first, accelerate the development of a large national unified market and increase the level of firm dual circulation coupling synergy. Second, explore the potential of domestic and international markets by expanding market demand. Improve the efficiency of resource allocation. Strive to improve the level of coupling synergy of dual circulation, and thus enhance the survival of firms. Third, as there are differences in the impact of dual circulation coupling synergy on the survival of firms, the government should take into account the heterogeneity of firms and industries when formulating policies.

## Supporting information

S1 Data (XLSX)
